# Ocular Movement Examination in Peripheral Vestibular Disorders as a Tool to Improve Diagnosis: A Literature Review

**DOI:** 10.3390/medicina60101665

**Published:** 2024-10-11

**Authors:** Gabriela Cornelia Musat, Calin Petru Tataru, Ovidiu Musat, Mihai Alexandru Preda, Mihnea Radu, Andreea Alexandra Mihaela Musat, Mihaela Roxana Mitroi

**Affiliations:** 1 ENT Department, “Carol Davila” University of Medicine and Pharmacy, 020021 Bucharest, Romania; gabriela.musat@umfcd.ro (G.C.M.); mihai.preda@umfcd.ro (M.A.P.); 2 Ophthalmology Department, “Carol Davila” University of Medicine and Pharmacy, 020021 Bucharest, Romania; calin.tataru@umfcd.ro; 3 Clinical Hospital Colentina, 020125 Bucharest, Romania; mihnea.radu96@yahoo.com; 4 Doctoral School, “Carol Davila” University of Medicine and Pharmacy, 020021 Bucharest, Romania; andreea-alexandra.musat@rez.umfcd.ro; 5 ENT Department, University of Medicine and Pharmacy Craiova, 200349 Craiova, Romania; mhlmitroi@yahoo.com

**Keywords:** nystagmus, vestibular disorder, peripheral vestibular nystagmus, ocular movements

## Abstract

*Background and Objectives*: This study reviews the current literature on ocular movements, specifically focusing on nystagmus associated with peripheral vestibular disorders, to enhance diagnostic accuracy. The evaluation of ocular movements, particularly nystagmus, provides essential insights into the function and dysfunction of the vestibular system, helping clinicians distinguish between peripheral and central causes of vertigo and imbalance. *Materials and Methods*: A comprehensive search of PubMed was conducted using key terms such as “ocular movements”, “nystagmus”, “vestibular nystagmus”, and “peripheral vestibular disorders”. *Results*: The search yielded 2739 titles, and after a rigorous selection process, 52 articles were reviewed in full. Discussion: The review highlights different classifications and types of nystagmus, including physiological and pathological forms, and their diagnostic relevance in vestibular disorders such as benign paroxysmal positional vertigo (BPPV), vestibular neuritis, and Meniere’s disease. Diagnostic techniques like video/electro-oculography are emphasized for their role in assessing vestibular function and identifying abnormalities. The study underscores the importance of detailed ocular examination in the diagnosis of peripheral vestibular disorders and proposes an algorithm to aid this process. *Conclusions*: While not a systematic review, this study highlights the importance of detailed ocular examination in diagnosing peripheral vestibular disorders and presents an algorithm to facilitate this process. It also emphasizes the need for continued research and advancements in vestibular medicine to further understand ocular movements and their clinical significance, ultimately contributing to improved patient outcomes.

## 1. Introduction 

The evaluation of ocular movements, especially nystagmus, is a valuable examination providing information about the peripheral or central vestibular system. Neurologists, ENT specialists, and ophthalmologists should be familiarized with the examination of ocular eye movements in order to diagnose different vestibular and neurological disorders. Among these, peripheral vestibular disorders stand out as a significant group wherein the examination of nystagmus offers invaluable diagnostic insights. The vestibular system, which encompasses the inner ear and its connections to the brain, plays a pivotal role in maintaining balance and spatial orientation. When its function is compromised, nystagmus often emerges as one of the earliest and most telling indicators.

There are four types of eye movements: smooth pursuit, saccades, vergence, and vestibulo-ocular movements. Nystagmus is a type of eye movement induced by the malfunction of the vestibulo-ocular reflex expressed as a repetitive eye movement with special characteristics. Nystagmus is defined as an involuntary, rapid rhythmic oscillatory eye movement [1]. The name nystagmus has a Greek etymology coming from the words nustagmos (drowsiness, nodding) and nystazein (to be sleepy or doze) [2,3]. There are multiple causes of nystagmus, and the pathophysiology of some types of nystagmus is still a subject of research.

Vestibular dysfunctions that occur in different vestibular disorders modify the function of the vestibulo-ocular reflex and generate nystagmus movements. The examination of nystagmus is of paramount importance for the diagnosis of vestibular disorders. Careful evaluation of nystagmus not only aids in localizing the site of the vestibular dysfunction but also helps in distinguishing between peripheral and central causes of vertigo and imbalance. Peripheral vestibular disorders, such as benign paroxysmal positional vertigo (BPPV), vestibular neuritis, and Meniere’s disease, each present with characteristic patterns of nystagmus. By understanding these patterns, clinicians can make more accurate diagnoses, tailor appropriate treatments, and improve patient outcomes.

Vertigo and dizziness are often encountered in the daily practice of general practitioners, neurologists, and otolaryngologists. Studies show that vestibular vertigo has a lifetime prevalence of 7.4%, a 4.9% one-year prevalence, and a 1.4% one-year incidence. It is three times more prevalent in the elderly and women [4,5]. A study published in 2024 established the fact that vestibular disorders affect an estimated 3% of the US population [6].

The main endpoint of this paper is to establish a diagnostic algorithm for peripheral vestibular disorders, with a particular focus on nystagmus. This article explores the significance of nystagmus in the diagnostic process, examining the underlying mechanisms, diagnostic techniques, and clinical implications. It emphasizes the importance of a thorough ocular movement examination within vestibular medicine. By presenting a focused diagnostic algorithm, the paper aims to enhance the diagnostic accuracy and clinical management of practitioners dealing with vestibular pathologies.

## 2. Materials and Methods

We conducted a comprehensive search in two major medical databases, PubMed and Embase, from January 2000 to December 2023. The search was restricted to articles published in English and human studies. The exact phrases and syntax used for the database query were as follows: “nystagmus”, “peripheral vestibular disorders”, “ocular movements”, “ocular movements nystagmus”, and “peripheral vestibular disorders nystagmus”. The inclusion criteria comprised original research articles, systematic reviews, and meta-analyses that focused on the diagnosis of vestibular disorders through the assessment of ocular movements. Exclusion criteria included single-patient case reports, which were considered as isolated occurrences from which no generalizable conclusions could be drawn, non-clinical studies, research focused on animal models, and papers with titles or abstracts related to central vestibular disorders. The search was updated in March 2024 to ensure the inclusion of the most recent literature, based on the same inclusion and exclusion criteria.

## 3. Results

**Figure 1:**
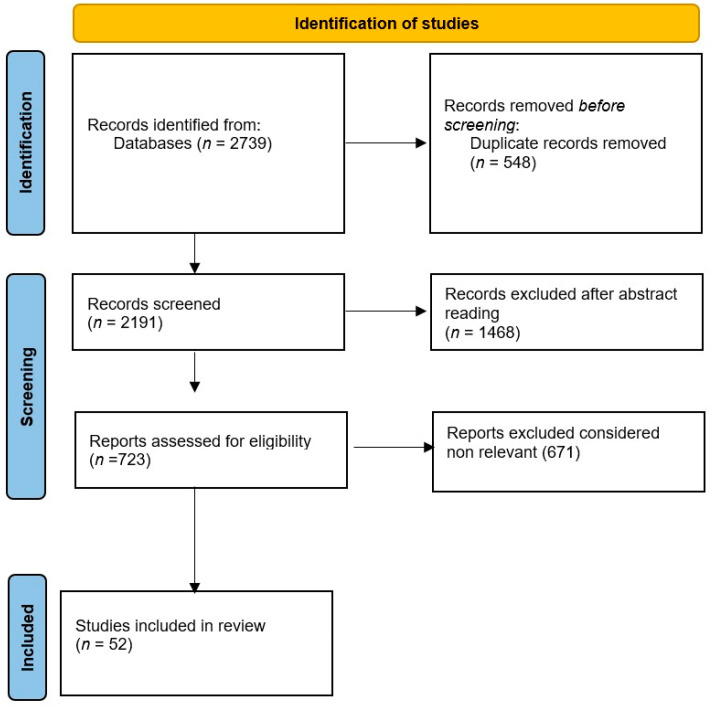
Flow diagram for the literature review on ocular movements induced by peripheral vestibular disorders as a tool to improve diagnosis.

The search initially identified 2739 titles (956 “nystagmus”, 446 “peripheral vestibular disorders”, 1114 “ocular movements”, 135 “ocular movements nystagmus”, 88 “peripheral vestibular disorders nystagmus”). After removing duplicates, 2191 unique titles remained. Abstracts were then reviewed, leading to the exclusion of 1468 titles. Papers without abstracts were not considered. Further evaluation of the remaining abstracts allowed for the exclusion of clinically irrelevant articles according to the established criteria. The relevant abstracts were subsequently assessed in full, resulting in 52 articles that were read in their entirety. The search results were evaluated by two groups of independent reviewers who evaluated the quality and relevance of each study. Any disagreements were resolved by consensus, and when necessary, a third reviewer was consulted in order to limit possible bias. We used a flowchart to organize the literature (see Figure 1). We have to state that this is not a systematic review because it was not possible to analyze the literature on this topic from a statistical point of view. Due to the heterogeneity of study designs and diagnostic tools, a systematic review was not feasible. Additionally, some studies lacked standardized reporting, further complicating a systematic approach.

To provide a more detailed explanation of the selection process, the following is a comprehensive list of inclusion and exclusion criteria:

1. Inclusion criteria:
  - Focus on peripheral vestibular disorders: Studies must specifically address peripheral vestibular disorders (e.g., benign paroxysmal positional vertigo, vestibular neuritis) and their association with ocular movements.
  - Diagnostic use of ocular movements: Only studies examining ocular movements (such as nystagmus, gaze stability, or smooth pursuit) as a diagnostic tool for peripheral vestibular disorders are included.
  - Human clinical studies: Studies involving human subjects and clinical investigations into vestibular disorders and ocular movement diagnostics were included.
  - Peer-reviewed articles: Only peer-reviewed journal articles are considered, ensuring methodological rigor and quality of research.
  - Full-text availability: Only studies with available full-text versions for a comprehensive review of methods and results were included.
  - Articles in English: Studies published in English were included for accessibility and consistency in evaluation.
  - Relevance to diagnostic improvement: Articles had to address how ocular movement examination contributed to improving diagnostic accuracy, speed, or clinical outcomes in peripheral vestibular disorders.
2. Exclusion criteria:
  - Single-patient case reports: Excluded to focus on more generalizable findings applicable to larger patient populations.
  - Non-clinical studies: Research involving theoretical frameworks, computational models, or non-clinical settings was excluded.
  - Animal studies: Studies focused on animal models were excluded to ensure the clinical relevance of the findings.
  - Studies on central vestibular disorders: Articles examining central vestibular disorders (e.g., brainstem or cerebellar issues) were excluded to maintain a focus on peripheral vestibular conditions.
  - Lack of abstract: Papers without abstracts or sufficient preliminary information to assess relevance were excluded.
  - Studies lacking diagnostic focus: Articles focusing purely on treatment, rehabilitation, or unrelated aspects of vestibular disorders (like lifestyle management) without diagnostic relevance were excluded.
  - Inadequate methodological rigor: Studies that lacked proper methodology, control groups, or statistical analysis were excluded based on low scientific rigor.
  - Non-standardized reporting: Articles that failed to provide clear data on diagnostic criteria, ocular movement measurement methods, or outcomes were excluded due to lack of transparency.

The studies included in this review covered a wide range of vestibular disorders. We summarized the key findings as follows:

- Benign paroxysmal positional vertigo (BPPV). A total of 15 studies have explored the diagnostic use of nystagmus in BPPV, 12 reported a high diagnostic accuracy when video-oculography was used. Common findings include nystagmus patterns that were specific, aiding the differential diagnosis.
- Meniere’s disease. Six studies focused on Meniere’s disease, with mixed results concerning the utility of ocular examination. While nystagmus can be observed in the acute attack, it is not a reliable diagnostic marker between episodes.
- Vestibular neuritis. Nine studies examined patients with vestibular neuritis, all reporting characteristic horizontal–torsional nystagmus in the acute phase. All the studies emphasized the fact that ocular examination was useful in differentiating peripheral central causes of vertigo.
- Chronic vestibular disorders. Four studies investigated chronic vestibular disorders, where nystagmus was not commonly observed, but ocular movements were still a valuable tool to assess the compensation phase in patients with vestibular loss.

## 4. Discussion

### 4.1. Different Classifications of Nystagmus Are Using Various Criteria

Considering the length of the two phases of nystagmus, it is described as a jerk nystagmus or a pendular nystagmus. The jerk nystagmus is typical in vestibular disorders, with a slow phase followed by a rapid one which defines the sense of the nystagmus [7]. The pendular nystagmus has two slow phases, often seen in congenital nystagmus [8], generated mainly by neurological demyelinating disorders, pharmacological intoxication, or other CNS causes [1,9,10].

For clinical purposes, nystagmus is considered in connection with the direction of the gaze in which it is elicited or increased, the conjugacy, the waveform, plane or planes of oscillation, amplitude, and frequency [8]. The nystagmus can be uni- or binocular. The direction can be oriented to the right, the left, upward, or downwards. The form of nystagmus can be linear, rotatory, or combined.

Barany Society established in 2019 a classification for physiological and pathological nystagmus. Physiological vestibular nystagmus comprises a multitude of eye movements elicited by the stimulation of a normal vestibule. Pathological nystagmus is a manifestation of a lesion either in the peripheral vestibular system or in the central nervous system.

Nystagmus must be differentiated from other eye movements such as: saccadic intrusions and oscillations, square wave jerks, ocular flutter, opsoclonus, spasmus nutans, superior oblique myokymia [1,11].

The cardinal symptom linked to nystagmus is oscillopsia which is a perception of oscillation of the visual environment, a subjective illusion of visual motion [12].

**Figure 2:**
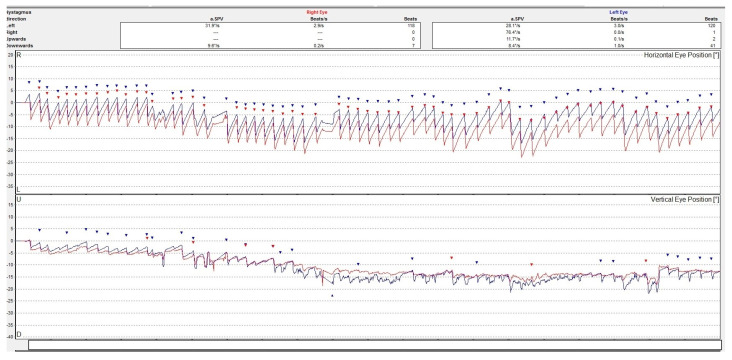
Video-oculography graph showing a horizontal-torsional nystagmus with the fast phase beating to the left in a patient with acute right peripheral vestibulopathy. The right eye movement is represented by a line colored in red and the left eye in blue. The dots represent the nystagmus secuses. The upper graph is for the horizontal and the lower one is for the vertical movements of the eyes. The torsional movement is decomposed in two directions.

Although a good examination of nystagmus and other eye movements can be performed by clinical examination, a better description is obtained by oculographic examination [8]. Electro- or video-oculography can provide objective information on ocular movements. The direction, amplitude, and slow phase velocity of the nystagmus are evaluated in a quantitative manner providing a comprehensive insight on the ocular movements. See Figure 2.

### 4.2. Relevant Anatomy and Physiology Elements

The peripheral vestibular system is represented by the inner ear, the vestibular ganglia, and the vestibular nerve. The inner ear has five sensory structures specialized in detecting head movements. There are three ampullar crests for each ear situated in the semicircular canals and two maculae in the utricle and the saccule. The maculae are specialized receptors specialized for sensing the linear movements of the head. The ampullar crests are receptors for the angular movements of the head. The cell bodies of the vestibular nerve are situated in the Scarpa ganglia located in the internal auditory canal [13,14]. The primary vestibular afferents synapse in the vestibular nuclear complex in the brainstem. Vestibular afferents from the vestibular nuclei relay signals to the extraocular motor nuclei, the spinal cord, and the cerebellum [15]. The neural links between the vestibular nuclei and extraocular muscles ensure the function of the vestibulo-ocular reflex.

To understand the mechanisms of nystagmus it is essential to understand the role of the visual system in maintaining the vision of the environment steady on the retina during head movements. This means that the line of sight originating from the fovea has to be held steady in order to keep the image clear and stable [16]. A gaze shift of more than 4 degrees per second generates oscillopsia, a perception of motion of the surrounding environment [17].

Three mechanisms ensure a steady view: fixation, the gaze-holding system, and the vestibular reflex. The fixation mechanism has two different components: one that prevents the image slip from the fovea triggering corrective movements, and another one that suppresses unwanted saccades. The gaze-holding system (neural integrator) is important in maintaining stable vision in an eccentric eye position. It is a mechanism that safeguards a constant level of muscle contraction to counteract the pull of the extraocular suspension structures [16,18].

Normal daily activities involve high velocities of head movements (up to 550 degrees per second), high accelerations (up to 6000 degrees per square second), and a frequency of up to 20 Hz. The vestibular system is the system developed in order to detect movements in this range of velocity, acceleration, and frequency. Normally both inner ears transmit an equal number of impulses through the vestibular nerves. When the head turns in one direction the number of impulses transmitted from that ear increases, and the number of impulses from the contralateral ear decreases. This is how the central nervous system is informed about the head movements, and this is how the ocular movements are triggered through the connections between the vestibular nuclei and the oculomotor nuclei. The vestibulo-ocular reflex is a reflex between the inner ear and the eye muscles with a very short latency (5–7 ms) guaranteeing clear vision during normal head velocities. The vestibular system is intimately related to the ocular system. During high-speed movements, the eyes move in the opposite direction, maintaining the image steady on the retina. The velocity of the eyes is equal to the velocity of the head [19]. The vestibulo-ocular reflex is a three-neuron chain reflex: a primary sensory afferent neuron from the Scarpa ganglia, a vestibular nucleus neuron in the brainstem, and an oculomotor neuron. The reflex is organized in such a manner that the vestibular nuclei on the same side as the excited labyrinth send excitatory projections to the oculomotor nuclei on the opposite side and inhibitory projections on the same side. This type of organization is easy to understand for the horizontal VOR elicited from the lateral semicircular canals. It involves contralateral abducens nucleus neurons and ipsilateral medial rectus neurons. The same organization is preserved for the vertical canals [20].

The vestibular end organs in the inner ear can be dysfunctional in different vestibular disorders. The inappropriate functioning can be manifested either by diminished/absent neural impulses to the vestibular nuclei, by hyperexcitation, or by unexpected variations of the activity. In essence, the vestibular organs are designed to inform the central nervous system about the movements of the head. Peripheral vestibular system abnormalities generate an asymmetry of the VOR leading to nystagmus.

Oculomotricity testing in the examination of a patient with vertigo or dizziness comprises a series of assessments. Typically, a clinician will assess five types of eye movements:

- smooth pursuit which keeps the image of the object in the fovea;
- saccadic which positions the image of the object over the fovea;
- optokinetic which is a response to image movement by generating slow pursuit movements followed by rapid refixation movements;
- vergence which is a movement of the eyes in opposite directions in order to maintain the image of the object on both foveas;
- vestibular movements which generate eye movements equal and opposite to the movements of the head [21].

The assessment of smooth pursuit, saccades, vergence, and optokinetic oculomotricity is a valuable tool to assess the integrity of the of central vestibular disorders [21]. Vestibular ocular motor screening (VOMS) is a tool created to evaluate patients post concussions. It includes the evaluation of smooth pursuit, saccades, and optokinetic nystagmus in relation to symptoms such as dizziness, fogginess, visual issues, and headache [22].

In our paper, we will focus on the modifications of ocular movements generated by the alteration of the VOR in cases of peripheral vestibular disorders.

### 4.3. Elements of Pathophysiology

In acute peripheral vestibulopathy, the neural input from the inner ear receptors to the vestibular nuclei is absent or reduced significantly. The lack of neural input on one side is reflected in the functionality of the vestibulo-ocular reflex and, as a direct consequence, a nystagmus with the fast phase toward the healthy ear is triggered.

In Meniere’s disease the function of the affected vestibular end organ is extremely variable in relation to different phases of the disease. The accepted pathophysiological theory explaining the symptoms of the disease is endolymphatic hydrops. Various other factors such as pressure changes, ionic disequilibrium, and endocochlear potentials are also discussed. The attack of Meniere is triggered by the increment in the pressure in the labyrinth followed by the rupture of the membranes that allow the mixture of the endolymph with the perilymph, paralyzing the receptors. The chemical mixture bathing the receptors leads to a depolarization blockade and transient loss of vestibular function on the affected side. This pathophysiological theory explains why the nystagmus in Menieres disease has different directions at different moments in the evolution of the disorder. At the beginning of the attack, on account of the excitation induced by the increment of the pressure in the labyrinth, the nystagmus is horizontal–rotatory of the excitation type beating to the affected ear. Afterwards, when the vestibular nerve receptors’ neural terminations are paralyzed as a consequence of ruptured membranes, the nystagmus beats to the unaffected ear, signalizing the hypofunction or total lack of function on that side [23].

In BPPV the otoliths dislodged in the semicircular canals induce unexpected endolymph flows in the canals. This can slow down or reverse the motion of the cupula generating signals incoherent with the actual head movement. The imbalance in the vestibular ocular reflex produced by incoherent sensory information induces vertigo and nystagmus.

Superior canal dehiscence pathophysiology is based on the existence of the third window in the otic capsule. This dehiscence induces an abnormal activation of the vestibular receptors produced by sounds or pressure. This abnormal excitation is manifested as vertigo and associated nystagmus [24,25].

### 4.4. Vestibular Nystagmus

#### 4.4.1. Physiological Vestibular Nystagmus

In this section, we include the ocular movements occurring as a consequence of the stimulation of a normal labyrinth. We consider physiological nystagmus responses the caloric nystagmus and the nystagmus during rotations of the head. Even though these types of nystagmus appear in a normal subject with a healthy labyrinth, their modifications can be used in assessing different vestibular disorders.

##### Caloric Nystagmus

**Figure 3:**
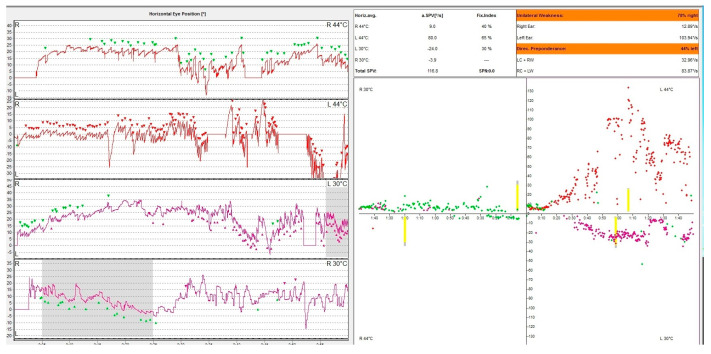
VNG graph. Caloric testing of a patient with acute unilateral vestibulopathy showing right caloric areflexia with left directional preponderance. The different colored dots represent nystagmus obtained following the warm and cold irrigation of each ear. Note that the right ear irrigation does not produce caloric nystagmus. The patient has a constant left beating represented in green unaffected by the caloric stimulation of the right ear.

Caloric nystagmus is a type of nystagmus elicited by a thermal stimulation of the vestibular end organ. In fact, it relies only on the stimulation of the lateral semicircular canal. Types of nystagmus are compared in caloric tests with air at 50 °C and 24 °C and with water at 44 °C and 30 °C. This test was described for the first time by Barany, an Austrian physician who won in 1914 the Nobel prize for this discovery [26]. The physiological explanation of this nystagmus is based on the fact that thermal stimulation induces a convection endolymphatic flow in the lateral semicircular canal, ampulopethal in the cold stimulation and ampulofugal in the cold stimulation. These convection currents deflect the ampullary crest thus triggering an action potential of this sensory receptor. This initiates the vestibulo-ocular reflex (VOR) generating the vestibular nystagmus. The warm water irrigation induces a slow movement of the eyes away from the side of the stimulus followed by a corrective fast saccade towards the side of the stimulus. The opposite happens with cold water irrigation. The vestibular response is calculated using the Jongkee formula [27]. Even though this physiological mechanism is accepted by the medical community there are aspects that cannot be explained. In an article published in 2019 Bell gives the hypothesis that maybe caloric response is in fact a response intermediated by the middle ear muscles, but the vestibular system has to be intact in order to register a positive response [28]. In Figure 3, we present the result of an oculography showing a right caloric areflexia.

##### Per-and Post-Rotational Nystagmus

**Figure 4:**
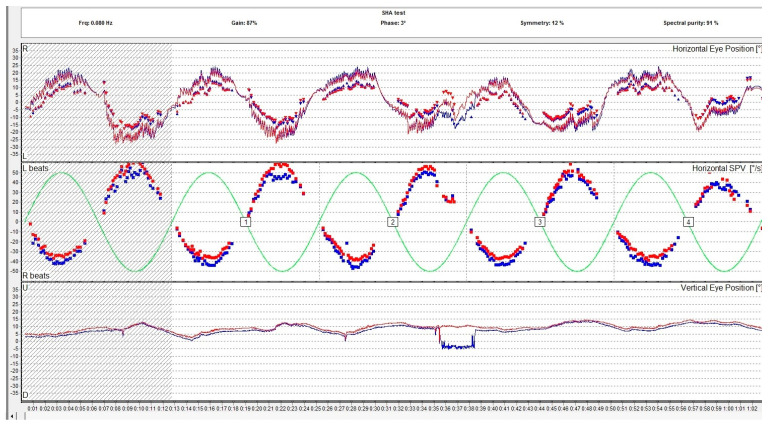
Rotational sinusoidal pendular test assessing the slow phase velocity of the per-rotational nystagmus compared to the rotary chair velocity. The report includes the measurement of the gain, the phase, and the symmetry. The red eye is represented in red, the left eye is represented in blue. The lines are representations of the eye movements and the dots represent the slow phase velocity.

Per- and post-rotational nystagmus of the head is another type of physiologic nystagmus. Although the specialized literature does not distinguish the two denominations, rotational nystagmus (a per-rotational or post-rotational nystagmus which is a horizontal nystagmus) should be differentiated from the form of rotatory nystagmus which is in fact a torsional nystagmus. In normal conditions, the vestibulo-ocular reflex (VOR) causes a tonic eye deviation, opposite to the head turn, designed to maintain fixation on an object. This action is counteracted by the saccades that originate from the frontal eye field in the opposite direction, which results in the fast component of the horizontal nystagmus as observed during rotations. This type of nystagmus is examined with a rotary chair. The rotational chair was introduced by Barany in 1907, and nowadays the ocular movements during rotation are registered using oculography and it is a part of the VNG testing along with the caloric and positional tests. Rotary chair testing has significantly evolved since it was first introduced and other rotational tests emerged. Nowadays, the battery of rotational tests includes sinusoidal testing, after-kinetic nystagmus, visual–vestibular interaction, and off-vertical axis rotation. Rotational tests evaluate both lateral semicircular canals at the same time. The rotary chair commonly evaluates frequencies in the range of 0.01–1.28 Hz. Gain, phase, and symmetry are the parameters used to assess the rotational nystagmus. Gain is the ratio of the amplitude of slow phase velocity of the rotational nystagmus to the amplitude of head movement. Phase is a parameter that describes the relationship between head movement and eye response. In Figure 4, we present the VNG graph of a sinusoidal pendular stimulation test, a rotational test used to assess the vestibular system.

Optokinetic nystagmus (OKN) is also a normal type, physiologic nystagmus, but this nystagmus is not produced with the implication of the vestibular system. OKN is a non-vestibular nystagmus in response to moving visual stimuli or surroundings. The slow phase has the same direction to the visual stimuli [29].

#### 4.4.2. Pathological Vestibular Nystagmus

This is a type of ocular movement produced by lesions in the peripheral (inner ear, VIII nerve) or central vestibular system. Examination of the eye movements can distinguish between the two forms of vestibular disorders [20]. There are special characteristics of the vestibular nystagmus. It is a jerk nystagmus meaning it has two phases: a phase of slow motion in which the eye drifts slowly and a rapid phase which is meant to refoveate the image.

The most frequent inner ear disorders producing nystagmus are vestibular neuritis, benign paroxysmal positional vertigo, Meniere’s disease, and superior canal dehiscence [30].

Different vestibular disorders induce a malfunction of the vestibulo-ocular reflex produced by the peripheral vestibular receptors transmitting corrupted information to the central nervous system thus causing abnormal ocular movements. Vestibular nystagmus is an ocular movement with special characteristics that can be induced by either central or peripheral vestibular disorders.

##### Spontaneous Peripheral Vestibular Nystagmus

Spontaneous peripheral vestibular nystagmus is a jerk nystagmus produced by an imbalance between the vestibular tone produced by the peripheral receptors. This nystagmus has several special characteristics: it is binocular and conjugate, has the same sense irrespective of the position of the eyes, obeys the law of Alexander (it is more intense when the eyes look in the direction of the fast phase) [31,32], and it is suppressed by visual fixation. It is the result of a lesion at the level of the peripheral vestibular system. In the case of a lesion of the entire labyrinth a spontaneous horizontal–rotatory nystagmus is elicited. Sometimes the torsional component is difficult to notice and the nystagmus seems to be horizontal. The tone imbalance between the two ears determines the direction of the fast phase always directed to the ear which is most active functionally. Acute vestibular neuropathy, labyrinthitis, acoustic neuroma, and most of the peripheral vestibular disorders have an inhibitory type of nystagmus [33], while Meniere’s disease can have an excitatory type [34,35]. It is difficult to determine whether nystagmus is of excitatory or inhibitory type only based on the characteristics of the eye movements. Other clinical or imagistic data must be corroborated in order to determine the type of spontaneous vestibular peripheral nystagmus.

##### Positional Nystagmus

Positional nystagmus is a nystagmus triggered by the change in position of the head with respect to gravity. It may be transient or persistent. Transient positional nystagmus is typical for benign paroxysmal positional vertigo (BPPV). Benign paroxysmal positional nystagmus (BPPN) is the nystagmus that appears in benign paroxysmal positional vertigo which is produced by displaced otoconia entering the semicircular canals. Whenever the head moves, a nystagmus is elicited. There are two varieties of benign paroxysmal positional vertigo differentiated by the position of the otoconia: free in the canal in canalolithiasis or on the cupula in cupulolithiasis. There are different types of nystagmus in BPPV corresponding to the involved canal: posterior, lateral, or anterior [36].

Benign paroxysmal positional nystagmus of posterior canal BPPV, canalolithiasis form is a nystagmus elicited by the Dix–Hallpike maneuver. It has a period of latency of a few seconds, and it lasts less than 1 min [37,38]. The nystagmus is a combination of torsional and vertical movements. The torsional component is a rotatory movement with the upper pole of the eye beating toward the lower ear and the vertical component beating upwards to the forehead. The torsional component of the nystagmus is more prominent in the lower eye and the vertical component is more important in the upper eye. If the gaze is directed to the lower eye the nystagmus seems to be more torsional and when the gaze is directed to the upper eye, the nystagmus seems to be more vertical. If fixation is not suppressed the nystagmus might seem torsional because the CNS is not very effective in suppressing torsional eye movements [39]. After the initial nystagmus is stopped a small reverse nystagmus might appear. The BPPN has a crescendo–decrescendo pattern. When returning to an upright position a nystagmus of the reverse direction happens.

The nystagmus in lateral canalolithiasis is a horizontal nystagmus elicited with no or very short latency in the supine roll position with the head rotated on both sides, with a geotropic direction on both sides, lasting under one minute [40]. Although the nystagmus is mainly horizontal, there is a small torsional component beating to the lower eye. The intensity of the nystagmus is increased with the head turned to the affected side. In lateral canal BPPV there is the possibility of triggering a nystagmus in the upright position, called pseudospontaneous nystagmus [41]. The mechanism of pseudospontaneous nystagmus in canalolithiasis is unclear. Some authors presumed that an otolith jam is implicated in the pathophysiology of this nystagmus [42]. In cupulolithiasis the mechanism of pseudospontaneous nystagmus seems to be explained by the cupular axis in the horizontal canal and the effect of gravity [41].

The nystagmus in cupulolithiasis of the horizontal canal is also a horizontal nystagmus beating apogeotropic in the supine roll position, on either side, with a brief or no latency, lasting more than one minute [43,44].

The nystagmus in canalolithiasis of the anterior canal is a down-beating vertical nystagmus elicited in one or both Dix–Hallpike positions, with a latency of one or a few seconds, with a duration of under one minute [45,46].

The nystagmus in posterior canal cupulolithiasis is elicited with no or brief latency by a “half Dix–Hallpike maneuver”, beating torsional with the upper pole of the eye to the lower ear and vertically upward, lasting more than one minute [47].

##### Headshaking-Induced Nystagmus

Headshaking-induced nystagmus is a nystagmus triggered by a movement of the head in the horizontal plane with a frequency of 2 Hz for 20 cycles. A nystagmus is elicited at the abrupt stop of the head movement. This nystagmus usually beats toward the better ear. The test is used to detect the unilateral peripheral deficit [48,49]. The pathophysiology of this nystagmus consists in an asymmetrical peripheral input and a central velocity storage mechanism.

##### Sound-Induced Nystagmus

Sound-induced nystagmus is nystagmus triggered by sounds and it is typical for superior canal dehiscence syndrome. The energy of the sound “leaks” toward the third window, causing movements of the endolymph that move the cupula, generating the nystagmus. The trajectory of this nystagmus is aligned with the plane of the canal that is involved [50].

##### Valsalva-Induced Nystagmus

Valsalva-induced nystagmus is typical for perilymph fistula and superior canal dehiscence syndrome as well as craniocervical junction abnormalities. The generating mechanism seems to be the increment in the intracranial pressure or the tympanic pressure causing direct deflection of the cupula [51].

##### Hyperventilation-Induced Nystagmus

Hyperventilation-induced nystagmus reveals the asymmetry in the functionality of the vestibular system, and it is positive in peripheral as well as central vestibulopathies. Subjects are instructed to take large breaths through the mouth for 30–90 s, and the nystagmus is observed without visual fixation. It beats toward the side with peripheral dysfunction. It may reveal a cerebellopontine angle tumor [52].

##### Pressure-Induced Nystagmus

Pressure-induced nystagmus is different from Valsalva-induced nystagmus, being triggered by extrinsic modifications of the pressures in the tympanic cavity. It is important to note the sense of the nystagmus in the case of negative or positive pressure in the external auditory canal. It is caused by the transmission of pressure from the external auditory canal, through the middle ear to the inner ear. It is typical for a labyrinthine fistula [53].

##### Vibration-Induced Nystagmus

Vibration-induced nystagmus is the nystagmus produced when a 100 Hz vibrator is placed on the skull. It beats toward the affected side in unilateral vestibular loss. The visual fixation should be blocked [54]. Table 1 provides a comprehensive summary of various types of vestibular nystagmus, detailing their descriptions, characteristics, and associated disorders. Each row corresponds to a specific type of nystagmus, offering a concise and informative overview that aids in understanding their clinical significance and diagnostic implications. This structured summary aids in the quick identification and understanding of various nystagmus types and their clinical contexts.

A special test designed to evaluate the functionality of the VOR is the head impulse test. This test is performed by rapidly moving the head while asking the patient to fix a target. A normal functioning peripheral vestibular organ will not affect the subject’s capacity to maintain the look on the target. A dysfunctionality of the VOR will affect the capacity to fix the view and to maintain the target over the fovea so a catch-up saccade will emerge. Corrective catch-up saccades usually appearing after the HIT usually indicate a peripheral vestibular hypofunction. In acute vestibular syndrome, normal clinical HIT should prompt the search for a central lesion [55,56].

### 4.5. Common Specific Vestibular Disorders and Their Association with Different Types of Nystagmus

The most frequent peripheral vestibular disorder is BPPV [57]. It accounts for 20–30% of the cases in specialized vestibular clinics [58]. It is defined by different distinguished types of nystagmus whose identification enables the diagnosis. As there are three semicircular canals, theoretically, each canal might be involved, so the diagnosis can be challenging. Identifying the canal is crucial as the treatment repositioning maneuvers must be addressed to the involved canal specifically in order to treat appropriately and avoid canal conversion. The main complaint of patients with BPPV is a short-duration (under 1 min) vertigo triggered by the modification of the position of the head in the gravitational field sometimes associated with nausea, vomiting, and instability. The diagnosis of BPPV relies on testing maneuvers intended to trigger the nystagmus associated with the disease. The classical maneuvers employed to assess the patient with a suspicion of BPPV are the Dix–Hallpike maneuver and the supine roll test maneuver. The Dix–Hallpike maneuver is intended to diagnose the posterior semicircular canal type of BPPV. After positioning the patient with the head turned 45 degrees toward the investigated ear in a supine position, a torsional nystagmus beating upward and to the undermost ear is triggered. This nystagmus appears after a short period of latency, has an increasing–decreasing pattern, and resolves in under one minute. When the patient returns to the upright position another nystagmus in the opposite sense appears. Repeating the maneuver has a diminishing effect on the nystagmus. Noticing these characteristics is important to correctly diagnose the variant of BPPV and exclude central positional nystagmus. The Dix–Hallpike test may trigger a nystagmus with different characteristics. If a horizontal nystagmus is triggered this should be an indication that the horizontal semicircular canal is involved. If a down-beating nystagmus is triggered that means it is a case of an anterior canal type of BPPV. Central positional nystagmus triggers different types of nystagmus with features that cannot be associated with any peripheral positional nystagmus.

Another frequent peripheral vestibular disorder is vestibular neuritis or acute unilateral vestibulopathy as the Barany Society recommends it to be named. The main symptom of acute unilateral vestibulopathy is a long-duration vertigo (days, weeks) associated or not with nausea, instability, or oscilopsia. The leading symptom is spontaneous nystagmus (usually horizontal–torsional), direction fixed, enhanced by the removal of visual fixation [59].

Meniere’s disease is characterized by episodes of vertigo, low to medium sensorineural hearing loss, and fluctuating aural symptoms (hearing, tinnitus, and a sensation of fullness) in the affected ear. The nystagmus in Meniere’s disease has a different duration according to the duration of the attack which can vary from 20 min to 12 h [60]. Between episodes, the physical exam of the patient with Meniere’s can be normal. During the attack, the nystagmus can vary in direction corresponding to the status of excitation or inhibition of the inner ear induced by the modifications of the pressure or the integrity of the membranes. The nystagmus is usually a horizontal–torsional nystagmus with a variable direction.

Superior canal dehiscence is not so frequent but it is worth knowing. It is characterized by symptoms like sound-induced vertigo, pressure-induced vertigo, tinnitus, and hyperacusis to bone-conducted sounds [61]. The physical exam may show sound- or pressure-induced nystagmus aligned with the affected semicircular canal involved.

**Figure 5:**
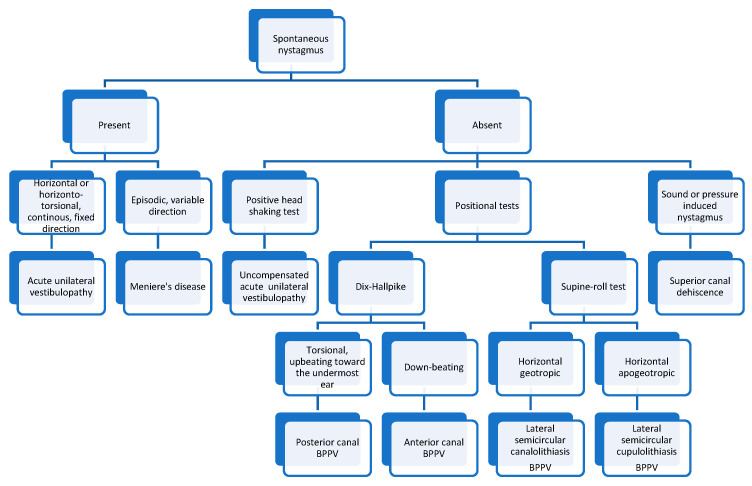
Algorithm for diagnosing vestibular disorders based on the nystagmus characteristics.

In Figure 5 we present an algorithm for diagnosing the most common vestibular disorders based on the nystagmus characteristics.

## 5. Conclusions

The evaluation of ocular movements, especially nystagmus, is important for diagnosing peripheral vestibular disorders because it brings essential insights into the function and dysfunction of the vestibular system. Through careful examination of ocular movements, clinicians can distinguish between peripheral and central causes of vertigo and imbalance, thus enabling accurate diagnoses and tailored treatments. This study underscores the importance of understanding the different types and characteristics of nystagmus, including physiological and pathological forms, and their implications for vestibular disorders such as benign paroxysmal positional vertigo, vestibular neuritis, and Meniere’s disease.

The review of the literature highlights the complex interplay between the vestibular and ocular systems, emphasizing the role of the vestibulo-ocular reflex in maintaining stable vision during head movements. Diagnostic techniques such as video/electro-oculography are important in assessing vestibular function and identifying abnormalities. By integrating these diagnostic tools with a thorough understanding of the underlying anatomy and physiology, healthcare professionals can enhance their diagnostics and improve patient outcomes.

In conclusion, the detailed examination of nystagmus and other ocular movements remains a cornerstone in the field of vestibular medicine. Continued research and advancements in diagnostic technologies are essential for furthering our understanding of nystagmus and its role in vestibular disorders. By fostering a deeper comprehension of these mechanisms, clinicians can better serve patients suffering from balance and spatial orientation issues, ultimately contributing to improved quality of life.

## Figures and Tables

**Table 1:** Key points and distinctions between different types of vestibular nystagmus and their clinical relevance.

Type of Nystagmus	Characteristics	Associated Disorders
Spontaneous peripheral vestibular nystagmus	Jerk nystagmus, binocular, conjugate, obeys Alexander’s law, suppressed by fixation, caused by peripheral vestibular imbalance	Acute vestibular neuropathy, labyrinthitis, acoustic neuroma, Meniere’s disease
Positional nystagmus	Triggered by head position change, transient or persistent	Benign paroxysmal positional vertigo (BPPV)
*Posterior canal canalolithiasis*	Elicited by Dix–Hallpike maneuver, latency of a few seconds, lasts less than 1 min, torsional and vertical components	BPPV
*Posterior canal cupulolithiasis*	Torsional and vertical nystagmus, elicited by half Dix–Hallpike maneuver, lasts more than 1 min	BPPV
*Lateral canal canalolithiasis*	Horizontal nystagmus, geotropic, elicited in supine roll position, lasts under 1 min	BPPV
*Lateral canal cupulolithiasis*	Horizontal nystagmus, apogeotropic, elicited in supine roll position	BPPV
*Anterior canal canalolithiasis*	Downbeating vertical nystagmus, elicited in Dix–Hallpike positions, lasts under 1 min	BPPV
**Headshaking-induced nystagmus**	Elicited by abrupt stop of headshaking, beats toward better ear	Unilateral peripheral deficit
**Sound-induced nystagmus**	Triggered by sounds, aligned with the plane of involved canal	Superior canal dehiscence syndrome
**Valsalva-induced nystagmus**	Triggered by increased intracranial or tympanic pressure	Perilymph fistula, superior canal dehiscence syndrome, craniocervical junction abnormalities
**Hyperventilation-induced nystagmus**	Reveals vestibular system asymmetry, beats toward side with peripheral dysfunction	Peripheral and central vestibulopathies, cerebellopontine angle tumor
**Pressure-induced nystagmus**	Triggered by pressure changes in tympanic cavity	Labyrinthine fistula
**Vibration-induced nystagmus**	Elicited by 100 Hz vibrator on skull, beats toward affected side	Unilateral vestibular loss

## Data Availability

All data are available upon request from the corresponding author.
